# Editorial: Role of sigma factors of RNA polymerase in bacterial physiology, volume II

**DOI:** 10.3389/fmicb.2023.1220519

**Published:** 2023-06-12

**Authors:** Miroslav Pátek, Riccardo Manganelli, Koichi Toyoda

**Affiliations:** ^1^Institute of Microbiology, AS CR, v. v. i., Prague, Czechia; ^2^Laboratory of Modulation of Gene Expression, Department of Molecular Medicine, University of Padua, Padua, Italy; ^3^Research Institute of Innovative Technology for the Earth, Molecular Microbiology and Biotechnology, Kyoto, Japan

**Keywords:** *Corynebacterium glutamicum*, *Mycobacterium tuberculosis*, *Listeria monocytogenes*, *Burkholderia*, pathogen, sigma factor, promoter, stress

Gene expression in bacteria is controlled by three main types of protein regulators: DNA-binding transcriptional regulators, two-component systems, and sigma (σ) factors of RNA polymerase. Since RNA polymerase is essential for transcription, sigma factors, as its subunits are involved in the control of each gene. In many cases, the main regulators control the expression of a gene cooperatively. Bacteria contain between two and more than 60 different σ factors, depending on the species. One of these, usually called σ^A^, is responsible for the transcription of most housekeeping genes, while the others are alternative. A large number of σ factors induced in most bacteria by various stresses belong to group 4 of sigma factors, called extracytoplasmic function sigma factors (ECF σ). All four articles in this Research Topic are dedicated to this type of σ. Two articles describe original research, and two others are reviews. Three contributions deal with gram-positive bacteria (*Corynebacterium glutamicum, Mycobacterium tuberculosis*, and *Listeria monocytogenes*) and one with a gram-negative bacterium (*Burkholderia* sp.).

*C. glutamicum* is the only exclusively nonpathogenic species in the selection. Busche et al. identified 12 genes belonging to the σ^E^ regulon. These genes were found to be involved in protein quality control, regulation of proteases, and maintenance of membrane integrity. Thus, σ^E^ was shown to regulate the cell surface response, as previously suggested. Interestingly, all identified σ^E^-controlled genes were also active with σ^H^. It has been shown in earlier studies that σ^H^ controls genes activated by heat and oxidative stress. The discovered overlap in the recognition specificity of σ^E^ and σ^H^ is based on very similar consensus sequences of σ^E^- and σ^H^-dependent promoters. It is evident that some proteins (e.g., chaperones, proteases, and oxidoreductases) that are essential under most stresses that lead to protein damage are alternatively induced by multiple sigma factors. Moreover, σ^H^ and σ^E^ drive the transcription of *sigB*, which also controls a variety of functions. Thus, σ^B^-σ^E^-σ^H^, which can be termed the “Big Three,” maintain the viability of *C. glutamicum* cells during the transition and stationary growth phases and under various types of stress conditions.

*Mycobacterium tuberculosis*, as a member of the Mycolata group, is related to *C. glutamicum*, but it is pathogenic. Therefore, σ^E^ may perform different functions in this bacterium exposed to host defense mechanisms. In their review, Manganelli et al. described σ^E^ as a master regulator in *M. tuberculosis*. It is involved in a regulatory network that controls responses to various environmental stresses (cell surface, pH and oxidative stress, and phosphate starvation) and functions connected to virulence. The expression of the *sigE* gene itself is controlled by three different sigma factors (σ^A^, σ^E^, and σ^H^) and a two-component system. In addition, the activity of σ^E^ is inhibited by the anti-sigma factor (similar to *C. glutamicum*), which is regulated by intracellular redox potential and proteolysis resulting from cell surface stress. Among several regulators, σ^E^initiates the transcription of σ^B^, which induces genes during the stationary growth phase. Thus, the activities and control mechanisms of σ^A^, σ^E^, and σ^H^ in *M. tuberculosis* are interwoven into a complex regulatory network similar to the tangle of *C. glutamicum*, although with different functions. Recently, this network was also found to be involved in the development of persister cells capable of surviving the action of various antimicrobials. In conclusion, σ^E^ is considered a master regulator in *M. tuberculosis*, whereas σ^H^ is considered a global regulator in the related *C. glutamicum*.

The importance of sigma factors in pathogenesis has also been documented in the genus *Burkholderia*, which includes several human pathogens. The very specific sigma factors described in the review by Grove have evolved in these bacteria as part of an “arms race” in which the pathogen and the host attempt to outwit the opponent by developing more sophisticated defense mechanisms. In the “battle for iron,” vertebrate hosts sequester iron in ferritin or the heme complex or reduce its levels by other mechanisms. As a result, the amount of free iron available to invading bacteria is greatly reduced, hindering pathogen replication and virulence. Several pathogenic *Burkholderia* species produce and secrete siderophores that efficiently chelate iron and allow its uptake into the cell. The production of siderophores and iron transporters is induced by iron limitation and controlled by specific ECF sigma factors. In some *Burkholderia*, the first genes in the gene clusters that determine siderophore synthesis and transport encode the ECF sigma factors MbaS (malleobactin sigma) and OrbS (ornibactin sigma). These genes are repressed by the global transcriptional regulator ferric uptake regulator (Fur) or other global regulators when iron is available. Thus, the cooperation of sigma factors and global regulators ensures that iron is accessible to the pathogen when its availability is limited.

*Listeria monocytogenes* is an intracellular foodborne pathogen that can infect both humans and animals. It can survive and grow under food production conditions, such as low and high temperatures, low and high pH, high salt concentrations, ultraviolet radiation, and in the presence of biocides. Alternative sigma factors have been found to be involved in the infection and replication cycles of this pathogen. In a comprehensive study of 15 mutants carrying different sigma factor deletions, Rukit et al. elucidated the role of each sigma factor in *L. monocytogenes* virulence. While σ^A^ is sufficient for the bacterium to invade and multiply in human cells, σ^B^ is essential for its invasion. Although all five *L. monocytogenes* sigma factors (σ^A^, σ^B^, σ^C^, σ^H^, and σ^L^) are involved in invasion and intracellular growth, interestingly, σ^B^ and σ^L^ can play a negative role. Deletion of σ^L^ can even enhance host invasion. In conclusion, the intricate regulatory network formed by sigma factors in *L. monocytogenes* requires further studies to unravel the mechanisms of host cell infections and replication within them. These findings may help improve the development of anti-Listeria agents needed to eliminate deadly listeriosis outbreaks.

## Author contributions

MP wrote the draft. RM and KT edited the text. All authors contributed to the article and approved the submitted version.

